# The Association of Gender in the Management and Prognosis of Vertebral and Sacral Chordoma: A SEER Analysis

**DOI:** 10.3390/jcm14051737

**Published:** 2025-03-04

**Authors:** Aladine A. Elsamadicy, Sumaiya Sayeed, Josiah J. Z. Sherman, Paul Serrato, Shaila D. Ghanekar, Sheng-Fu Larry Lo, Daniel M. Sciubba

**Affiliations:** 1 Department of Neurosurgery, Yale University School of Medicine, 333 Cedar Street, New Haven, CT 06510, USA; sumaiya.sayeed@yale.edu (S.S.); shaila.ghanekar@yale.edu (S.D.G.); 2 Department of Neurosurgery, University of Miami Miller School of Medicine, Miami, FL 33136, USA; josiah.sherman@yale.edu; 3 Department of Neurosurgery, Zucker School of Medicine at Hofstra, Long Island Jewish Medical Center and North Shore University Hospital, Northwell Health, Manhasset, NY 11030, USA; larrylo@northwell.edu (S.-F.L.L.); dsciubba1@northwell.edu (D.M.S.)

**Keywords:** chordoma, survival, spine tumor

## Abstract

**Background/Objectives:** Chordomas are rare primary osseous tumors of the spine and skull base that may portend significant morbidity and mortality. Gender disparities in the management and outcomes of spinal and pelvic chordomas have been sparsely studied. This study aimed to examine the effect of gender on the treatment utilization and outcomes in patients with vertebral column and sacrum/pelvis chordomas. **Methods:** A retrospective cohort study was performed using the 2000 to 2020 Surveillance, Epidemiology, and End Results (SEER) Registry, a U.S. population-based cancer registry database. Patients with histologically confirmed chordoma of the vertebral column or the sacrum/pelvis were identified using ICD-O-3 codes. The study population was divided into gender-based cohorts: male and female. The patient demographics, tumor characteristics, treatment variables, and mortality were assessed. **Results:** A total of 791 patients were identified and stratified by gender: 485 (61.3%) male and 306 (38.7%) female. The mean tumor size was similar between the cohorts (*p* = 0.377), as was the tumor location, with most arising from the pelvic bones/sacrum/coccyx (*p* = 0.953). While the treatment characteristics did not significantly vary, among patients who received both radiotherapy and surgery, neo-adjuvant radiotherapy was utilized at higher frequencies in the male patients (*p* = 0.011). For vertebral column chordomas, the median (*p* = 0.230) and five-year survival (*p* = 0.220) was similar between cohorts, and gender was not a predictor of survival (*p* = 0.239). Similarly, for pelvic chordomas, the median (*p* = 0.820) and five-year survival (*p* = 0.820) was similar between cohorts, and gender was not associated with survival (*p* = 0.816). **Conclusions:** Our study suggests that gender may influence treatment utilization but not mortality in patients with chordomas of the spine and sacrum.

## 1. Introduction

Chordoma is a rare subtype of primary osseous neoplasms of the spine and skull base believed to arise from remnants of the fetal notochord [1]. Chordomas are typically slow-growing yet locally invasive tumors with metastatic potential and high recurrence rates and may be associated with significant morbidity and mortality. Chordomas are most often seen in middle-aged patients, though they have been observed in children and the elderly [1,2,3]. While the overall incidence is noted to be 0.08 in 100,000 [4], some studies suggest a gender-based difference in the incidence of chordoma, with these tumors being slightly more common in males (58% of all chordomas) [5]. Unlike other primary bone tumors which can arise from the spine, such as osteosarcomas, chondrosarcomas, and Ewing’s sarcomas, chordomas are seen almost exclusively in the midline axial skeleton, from the clivus to the sacrococcygeal region, with many studies reporting sacrococcygeal chordomas as being the most common (30–60%), followed by the clivus/spheno-occiptal region (25–35%) and the mobile spine (15–30%), though some studies have found a near-equal distribution between these three major locations [1,2,6,7]. The initial presenting symptoms are dependent on the tumor location and can include headaches, myelopathies, bowel and bladder dysfunction, dysphagia, and airway dysfunctions [4]. As chordomas progress, their symptomatic burden includes chronic pain, depression, anxiety, and fatigue, which can greatly impact patients’ independence and quality of life [8,9]. Chordomas often have late diagnoses and a delay in care, which further exacerbates the disability and psychological burden for patients and their caregivers [8]. Treatment typically consists of en bloc/maximal surgical resection with clear margins in addition to high-dose, conformal neo-adjuvant or adjuvant radiotherapy [10,11,12]. While the five-year survival is similar between chordomas in the three major anatomic locations (mobile spine: 68.4%; skull base: 65%; sacrum 60%), the median overall survival is the greatest in patients with chordomas of the skull base (162 months), followed by those of the mobile spine (95 months) and the sacrum (87 months) [1], suggesting differences in the patient population, natural course, and treatment strategies between chordomas across these sites.

A patient’s gender has been shown to impact the incidence, treatment, and outcomes across many neurosurgical pathologies. For example, meningiomas are known to be more common in female patients, likely due to the high expression of progesterone estrogen receptors in these tumors [13]; however, the current literature largely suggests that a patient’s gender does not independently influence the management and outcomes in patients with meningiomas [14,15]. Researchers have examined the presence and impact of gender disparities among patients with other brain tumors [16], cerebral aneurysms [17,18,19], surgical epilepsy [20], degenerative disease of the spine [21,22,23], spine metastases [24], and intramedullary spinal cord tumors [25]. Some authors have studied the effect of the social determinants of health on patients with primary osseous tumors of the spine [26,27]. However, there remains a dearth of literature on the existence of gender disparities in patients with chordomas. Given the significant morbidity and mortality associated with chordomas, as well as the well-studied impact of gender disparities in other neurosurgical pathologies, it is warranted to assess the presence of gender disparities in the management and outcomes of chordoma.

The aim of this study was to assess the impact of patient gender on the treatment utilization and mortality among patients with chordoma. We hypothesized that there would be gender disparities in the use of treatment and mortality in chordoma patients.

## 2. Methods

### 2.1. The Data Source and Patient Population

The Surveillance, Epidemiology, and End Results (SEER) Program database is a set of national registries from the National Cancer Institute containing approximately 50% of the United States population. The November 2022 SEER dataset, consisting of 18 registries from 2000 to 2020, was queried to identify patients diagnosed with spinal chordoma (ICD-O: 9370) with primary sites at the vertebral column (ICD-O: 412) or the sacrum/pelvis (ICD-O: 414). The study population was further divided into two cohorts based on patient gender: male and female.

Exemption from Institutional Review Board approval was granted, as the patients in the SEER database were de-identified. Tumor histology was recorded using the World Health Organization (WHO) International Classification of Diseases for Oncology 3rd Edition (ICD-O-3) [28].

### 2.2. Data Collection

Demographic information on age (18 years and older), sex (male or female), race (White, Black, Hispanic, Other), median household income, marital status, and treating hospital location was analyzed for all patients. The tumor characteristics included size, defined as length of the greatest dimension and location. The treatment utilization included receipt of a surgical procedure, chemotherapy, or radiation therapy. Surgical resection was categorized as gross total resection (GTR) or partial resection. Chemotherapy was divided into no chemotherapy, chemotherapy only, or chemotherapy and surgery. Radiation therapy was characterized as not received, radiation only, or radiation and surgery, and it was further described by chronicity (radiation before or after surgery).

Parametric data were expressed as means ± standard deviations (SDs) and compared via a two-sided independent *t* test. Nonparametric data were expressed as medians (interquartile ranges [IQRs]) and compared via the Mann–Whitney U test. Categorical data were compared using the x^2^ test. The primary outcome of interest was overall survival. We constructed Kaplan–Meier (KM) survival curves for the two tumor locations (the vertebral column and pelvis), which were compared by calculating the median survivals. The KM model yielded hazard ratios (HRs) with 95% confidence intervals (95%CIs) and *p*-values using log rank (Mantel–Cox) tests. Data queries were performed using SEER*Stat, version 8.4.1.1. The statistical analysis was performed using RStudio (Posit PBC, Boston, MA, USA), version 2023.03.0+386, and GraphPad Prism (Dotmatics, Boston, MA, USA), version 9.5.1 (528).

## 3. Results

### 3.1. Patient Demographics

A total of 791 patients were identified, of whom 485 (61.3%) were in the male cohort and 306 (38.7%) were in the female cohort; see Table 1. The mean patient age was similar between the cohorts ([mean ± SD] male: 61.26 ± 15.20 years old vs. female: 61.04 ± 17.72; *p* = 0.858); see Table 1. The racial distribution of the patients was similar between the cohorts, with most of the patients in both cohorts being non-Hispanic White (male: 77.0% vs. female: 72.4%; *p* = 0.398); see Table 2. The income distribution was similar between cohorts ([≥USD 75,000 annual income] male: 49.5% vs. female: 49.7%; *p* = 0.917), though a greater proportion of patients in the male cohort were married compared to the female cohort (male: 68.4% vs. female: 54.3%; *p* < 0.001); see Table 1. The hospital locations were similar between cohorts, with most of the patients being in metropolitan areas with populations > 1 million (male: 62.7% vs. female: 64.1%; *p* = 0.793); see Table 1.

### 3.2. Tumor Characteristics

The mean tumor size was similar between cohorts (male: 75.07 ± 44.57 mm vs. female: 71.73 ± 43.85 mm; *p* = 0.377), as was the tumor location, with most arising from the pelvic bones/sacrum/coccyx in both cohorts (male: 57.7% vs. female: 57.5%; *p* = 0.953); see Table 1.

### 3.3. Treatment Characteristics

**Figure 1:**
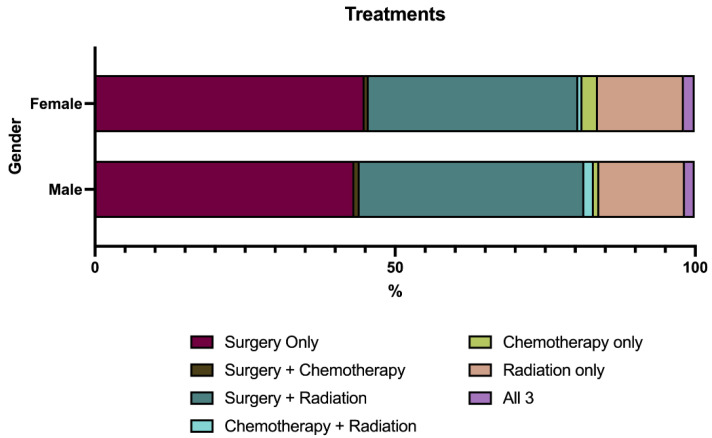
Treatment regimen utilized by patients.

The treatment characteristics were largely similar between cohorts; see Table 2 and Figure 1. Most of the patients in both cohorts underwent surgical treatment (male: 73.4% vs. female: 73.2%; *p* = 0.951); see Table 2. Among the patients who underwent surgery, GTR was performed at a similar rate in the patients of both cohorts (male: 81.2% vs. female: 80.1%; *p* = 0.743); see Table 2. Chemotherapy was infrequently utilized in both cohorts at similar rates (male: 4.5% vs. female: 5.2%; *p* = 0.656); see Table 2. Radiotherapy was commonly used across the study population, though it was utilized at similar rates between cohorts (male: 48.5% vs. female: 46.1%; *p* = 0.516); see Table 2. Among the patients who received both radiotherapy and surgery, neo-adjuvant radiotherapy was utilized in a greater proportion of patients in the male cohort compared to that in the female cohort (male: 13.2% vs. female: 7.9%; *p* = 0.011); see Table 2.

### 3.4. Survival Analysis—The Vertebral Column

**Figure 2:**
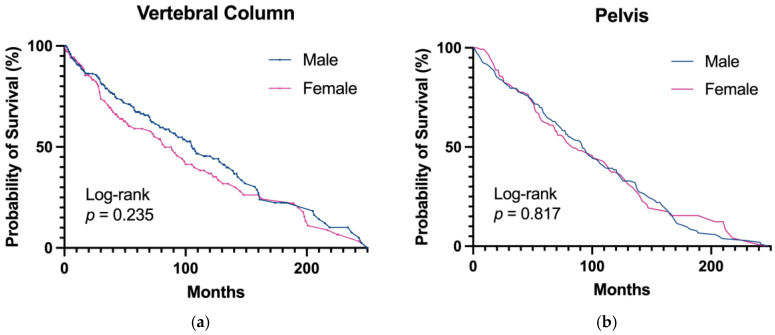
Survival analyses for patients with chordomas of the (**a**) vertebral column and (**b**) pelvis.

Among the patients with chordomas located in the vertebral column, the median survival ([median (IQR)] = male: 106 [87, 139] vs. female: 83 [58, 119]; *p* = 0.230) and five-year survival (male: 33% vs. female: 41%; *p* = 0.220) were similar between cohorts; see Table 3 and Figure 2a.

**Figure 3:**
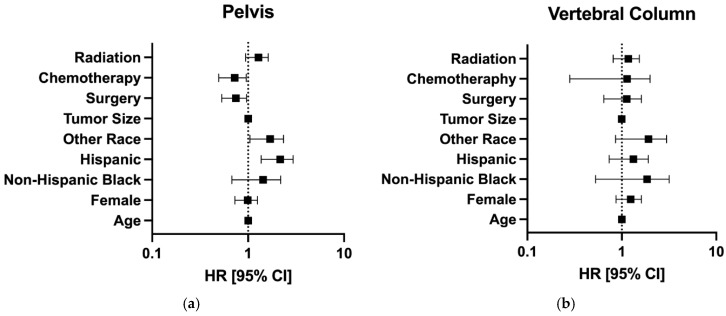
Hazard ratios of various factors among patients with chordomas of the (**a**) vertebral column and (**b**) pelvis.

Patient gender was not a risk factor for survival [(Female) HR: 1.20, 95%CI: 0.89–1.63, *p* = 0.239]; see Table 4 and Figure 3a. None of the other queried factors were associated with survival, including age [HR: 1.00, 95%CI: 0.99–1.01, *p* = 0.783], race [(Non-Hispanic Black) HR: 1.54, 95%CI: 0.71–3.30, *p* = 0.272], tumor size [HR: 1.00, 95%CI: 0.99–1.00, *p* = 0.440], surgery utilization [HR: 1.06, 95%CI: 0.68–1.64, *p* = 0.796], chemotherapy utilization [HR: 0.92, 95%CI: 0.41–2.08, *p* = 0.841], and radiotherapy utilization [HR: 1.14, 95%CI: 0.83–1.55, *p* = 0.414]; see Table 4 and Figure 3a.

### 3.5. Survival Analysis—The Pelvis

Among the patients with chordomas located in the pelvis, the median survival ([median (IQR)] = male: 92 [78, 107] vs. female: 85 [69, 116]; *p* = 0.820) and five-year survival (Male: 33% vs. Female: 37%, *p* = 0.820) were similar between cohorts; see Table 5 and Figure 2b.

Patient gender was not associated with survival [(Female) HR: 0.97, 95%CI: 0.74–1.26, *p* = 0.816]; see Table 6 and Figure 3b. The factors associated with survival in the patients with pelvic chordomas included Hispanic race [HR: 2.06, 95%CI: 1.42–2.99, *p* < 0.001], which was a positive predictor of mortality, and the utilization of surgical treatment [HR: 0.73, 95%CI: 0.54–0.97, *p* = 0.031], which was a negative predictor of mortality; see Table 6 and Figure 3b. The other queried factors were not associated with survival, including patient age [HR: 1.00, 95%CI: 0.99–1.01, *p* = 0.652], tumor size [HR: 1.00, 95%CI: 1.00–1.00, *p* = 0.555], chemotherapy utilization [HR: 0.92, 95%CI: 0.47–1.79, *p* = 0.803], and radiotherapy utilization [HR: 1.25, 95%CI: 0.96–1.63, *p* = 0.092]; see Table 6 and Figure 3b.

## 4. Discussion

In this retrospective SEER database study of 791 patients with chordomas of the vertebral column and the pelvis, patient gender was not associated with many of the patient- and hospital-level factors, tumor characteristics, or overall survival. The treatment characteristics were largely similar between the male and female cohorts, though neo-adjuvant radiotherapy was significantly more common among males compared to females. The median and five-year survival did not differ significantly between cohorts, nor was gender a predictor of the overall survival in vertebral and pelvic chordomas.

Patient gender is known to influence the incidence and outcomes of a number of tumors that occur in both male and female patients. In a 1973–2003 SEER database study of 827 patients with primary osseous neoplasms of the spine (including 215 patients with chordomas), Mukherjee et al. observed that 38% of these patients were female [29]. Similarly, in a 2004–2015 National Cancer Database study of 1266 patients with spinal and sacral chordomas, Wright et al. found that 37.5% of these patients were female [30]. A similar distribution has been reported in other studies [31,32,33,34]. Interestingly, some studies have demonstrated a varied gender distribution in patients with cranial chordomas. For example, in a 2004–2014 National Program of Cancer Registries database study of 3670 patients with chordomas, Das et al. found that the male-to-female incidence rate ratio (IRR) among patients with cranial chordomas was 1.30 (about 44% female) compared to the spinal chordoma male-to-female IRR of 1.70 (about 37% female) [34], suggesting that cranial chordomas affect a greater proportion of females compared to spinal/sacral chordomas. Nonetheless, most studies, examining both cranial and spinal/sacral chordomas, report a higher incidence of chordomas in male patients compared to that in female patients. In the present study of 791 patients with chordomas of the vertebral column and pelvis, 38.7% of the patients were female, similar to the incidence observed in previous studies. Understanding how patient gender influences disease incidence is necessary to accurately assess the impact of disparities in the treatment and outcomes in patients with chordomas.

Some authors have studied the impact of patient gender on the tumor characteristics among patients with chordomas. Tumor differentiation is used as an indicator of aggressiveness in patients with chordomas, as dedifferentiated chordomas exhibit genetic distinctions compared to conventional chordomas and are more clinically aggressive. In a retrospective cohort study of 79 patients with conventional and dedifferentiated chordomas who were treated at a high-volume cancer center, Nachwalter et al. observed that female patients accounted for 29% of conventional chordomas and 45% of dedifferentiated chordomas [35]. The relationship between anatomic location and patient gender has also been assessed. In a 1973–1995 SEER database study of 400 patients with chordomas, McMaster et al. found that while the anatomic location of the chordomas was near evenly distributed between the cranial, spinal, and sacral sites across males and females, when specifically examining the distribution among female patients, cranial chordomas were more common, sacral chordomas were less common, and spinal chordomas were nearly as common in female patients compared to male patients [6]. Similarly, in a 1973–2013 SEER database study of 1616 patients with cranial, spinal, and sacral chordomas, Zuckerman et al. found that skull base chordomas were overrepresented among female patients (45.1% vs. 41.1% overall), sacral chordomas were less common in female patients (28.7% vs. 31.4% overall), and chordomas of the mobile spine occurred at similar rates between female patients and the overall population (26.2% vs. 27.5% overall) [5]. Additionally, in a 2000–2018 SEER database study of 1536 cases of skull base and spinal chordomas, Vuong et al. found that chordomas of the skull base were slightly overrepresented among female patients (46.8% vs. 43.4% overall), while spinal chordomas were slightly underrepresented among female patients compared to the overall chordoma study population (53.2% vs. 56.6% overall) [36]. Conversely, in a multi-institutional study of 151 patients who underwent treatment for clival or spinal/pelvic chordomas, Ghaith et al. found that the anatomic location of chordoma was not greatly impacted by patient gender, with similar rates for clival (female: 40.0% vs. overall: 38.4%), spinal (female: 31.7% vs. overall: 31.8%), and sacrococcygeal chordomas (female: 28.3% vs. overall: 29.8%) [37]. In the present study, we found that anatomic locations of chordomas at the spine and the sacrum/pelvis were similar between the male and female cohorts, with most of the tumors in both cohorts occurring in the pelvic bones, sacrum, and coccyx (male: 57.7% vs. female: 57.5%; *p* = 0.953). While it is necessary to understand whether patient gender influences the tumor characteristics given that these characteristics may influence the treatment approaches and outcomes, it appears that tumor location is not significantly influenced by patient gender in patients with chordomas of the vertebral column and sacrum/pelvis.

Given the large impact that treatment utilization may have on the outcomes of patients with chordomas and other primary osseous tumors of the spine, some authors have tried to determine the influence of gender on the management employed for these patients. In a 1975–2016 SEER database study of 1035 patients with spinal chordomas, Zhang et al. reported that patient gender was not significantly associated with the utilization of surgery, radiotherapy alone, or both surgery and radiotherapy (*p* = 0.703) [38]. Similarly, in a 1975–2018 SEER database study of 263 patients who underwent GTR of spinal chordomas, Gendreau et al. showed that the utilization of radiotherapy following GTR occurred at similar rates between male and female patients ([male] no radiotherapy: 59.9% vs. radiotherapy: 69.4%; *p* = 0.388) [39]. Furthermore, in a 2004–2010 National Cancer Database study of 282 patients with vertebral or sacral chordoma, Yolcu et al. reported that patient gender did not significantly influence the receipt of GTR alone or the receipt of both GTR and radiotherapy [40]. Treatment utilization by patient gender was also assessed in the present study. We found that the treatment utilization was largely similar between the male and female cohorts, with most of the patients in both cohorts undergoing surgery, particularly GTR. The utilization of chemotherapy was low among both male and female patients (male: 4.5% vs. female: 5.2%; *p* = 0.656), which was expected given the low efficacy of chemotherapy in the treatment of chordoma. The use of radiation occurred at similarly high rates between the male and female cohorts (male: 48.5% vs. female: 46.1%; *p* = 0.516). These findings are in line with the treatment consensus for spinal and sacral/pelvic chordoma endorsing a combined treatment strategy of surgical excision and radiotherapy for many patients. Where the treatment utilization varied between cohorts, however, was in the timing of radiotherapy in relation to surgery. Neo-adjuvant radiotherapy was utilized at higher frequencies in the male cohort compared to that in the female cohort. Additionally, a higher proportion of patients in the female cohort underwent a non-standard radiation sequence compared to the patients in the male cohort, who were overall more likely to undergo a standard radiation sequence, such as adjuvant or neo-adjuvant radiation. Given the high importance of radiotherapy in the effective management of many chordomas, it is warranted to explore the ideal radiotherapy timing and treatment strategies and whether the increased frequency of non-standard radiotherapy in female patients influences patient outcomes.

Several studies have assessed how complications and mortality are impacted by gender in patients with chordomas of the vertebral column and the sacrum/pelvis. In a study of 26 patients (13 males and 13 females) who underwent surgical resection for chordoma of the mobile spine at a single tertiary care center from 1990 to 2015, Kolz et al. reported that patient gender was not independently associated with the incidence of post-operative infection [(male gender) HR: 1.47, 95%CI: 0.26–8.05, *p* = 0.65] or hardware failure [HR: 1.33, 95%CI: 0.24–7.30, *p* = 0.73] [41]. Furthermore, the authors found that patients being male was not associated with their disease-specific survival [HR: 2.98, 95%CI: 0.61–14.43, *p* = 0.17] or metastasis-free survival [HR: 5.99, 95%CI: 0.74–48.10, *p* = 0.09] but was a risk factor for local recurrence-free survival [HR: 8.85, 95%CI: 1.12–69.63, *p* = 0.03] [41]. Additionally, in a 1975–2018 SEER database study of 263 patients with spinal chordomas who underwent GTR, Gendreau et al. found that patient gender was not an independent risk factor for overall survival [(male gender) HR: 0.83, 95%CI: 0.69–1.47, *p* = 0.231] [39]. Interestingly, in a review of 682 patients with spinal chordomas, including 108 studies as well as 30 patients treated at a single center, Zhou et al. found that the median progression-free survival was significantly greater among female patients compared to that in male patients (83 months vs. 62 months in males; log rank *p* = 0.076), though the median overall survival was similar between the male and female patients (120 months vs. 113 months in males; log rank *p* = 0.631) [42]. In a 2004–2010 National Cancer Database study of 282 patients with vertebral and sacral chordomas who underwent surgical resection with or without radiotherapy, Yolcu et al. found that patient gender was not a significant risk factor for overall survival [(male gender) risk ratio: 1.092, 95%CI: 0.582–2.049, *p* = 0.783] [40]. Other studies evaluating the impact of patient gender on survival in the vertebral column/sacral chordoma population have yielded comparable findings [31,33,43]. In the present study, patient gender was not a significant risk factor for survival in patients with vertebral column or pelvic chordomas, in line with most existing studies. Interestingly, most of the studies used mortality/survival as the primary endpoint without reporting the influence of patient gender on non-death complications such as hardware infection or wound disruption after surgery or radiotherapy, possibly due to the limitations of the commonly used datasets. While it appears that patient gender has a minimal effect on mortality in the spinal/sacral chordoma population, it is warranted to explore how patient gender affects non-death endpoints such as post-operative complications.

This study should be evaluated in the context of limitations which may have implications in terms of its interpretation and generalizability. First, the analysis was retrospective, with data available only according to ICD diagnosis codes, which may have contained coding and reporting biases. Given the retrospective nature of this study, some variables that may have impacted the outcome measures were included, as they were not recorded in the SEER database. For instance, baseline characteristics like comorbidities and perioperative variables such as the use of instrumentation or post-operative adverse events could provide key insights into patient outcomes but are not provided in the database. In addition, there is a possibility of misclassified, incorrectly recorded, or incomplete data inherent to the use of a large database. Despite the aforementioned limitations, this study is one of the first to evaluate the impact of patient gender on treatment and mortality in spinal and pelvic chordoma patients using a relatively large sample size, especially given its low incidence. While past studies have focused on epidemiological trends, our study emphasizes the role of gender in the treatment and outcomes for chordoma, a potential link that has not been sufficiently explored in the existing literature. While the results do not suggest that gender greatly influences survival, our study can be used as a foundation for further studies investigating this potential relationship. Future studies should focus on expanding the sample size and utilizing a variety of databases to assess the presence of gender disparities across multiple cross-sections of the chordoma patient population.

## 5. Conclusions

Our study suggests that patient gender may play a role in treatment utilization but does not impact mortality in patients with chordomas of the spine and sacrum. Patient gender did not significantly influence tumor size or site. Aside from non-standard radiation sequences being utilized more frequently in female patients, the treatment utilization was similar between male and female patients. Finally, patient gender was not a significant risk factor for survival in patients with chordomas of the vertebral column and the sacrum/pelvis. Further studies are indicated to explore the impact of patient gender on non-death complications among patients treated for chordoma.

## Figures and Tables

**Table 1:** Demographic information.

Variable	Male (*n* = 485)	Female (*n* = 306)	*p*-Value
Age (years)			
Mean ± SD	61.26 ± 15.20	61.04 ± 17.72	0.858
Median [IQR]	63 [52, 73]	63 [51, 75]	0.717
Age distribution			0.390
18–49	22.3	23.5	
50–69	44.1	39.2	
70+	33.6	37.3	
Race (%)			0.398
Non-Hispanic White	77.0	72.4	
Non-Hispanic Black	2.7	4.3	
Hispanic	11.4	12.2	
Other	8.9	11.2	
Income (%)			0.917
<USD 65,000	25.4	24.2	
USD 65,000–74,999	25.2	26.1	
≥USD 75,000	49.5	49.7	
Marital status			<0.001 *
Single	31.6	45.7	
Married or domesticpartner	68.4	54.3	
Hospital location (%)			0.793
Metropolitan, >1M	62.7	64.1	
Metropolitan, <1M	29.9	27.8	
Rural	7.4	8.2	
Tumor size (mm)			
Mean ± SD	75.07 ± 44.57	71.73 ± 43.85	0.377
Median [IQR]	67.00 [41.00, 100.00]	64.00 [40.00, 93.00]	0.307
Site (%)			0.953
Vertebral column	42.3	42.5	
Pelvic bones, sacrum, coccyx	57.7	57.5	

SD: standard deviation; IQR: interquartile range; mm: millimeters; 1M: 1 million. * statistically significant.

**Table 2:** Treatments.

Variable	Male (*n* = 504)	Female (*n* = 318)	*p*-Value
Surgery (%)			
Received	73.4	73.2	0.951
Surgical procedure (%)			0.743
Partial resection	18.8	19.9	
Gross total resection	81.2	80.1	
Surgical excision (%)			0.353
Partial	18.8	19.9	
Local	28.7	33.6	
Radical	52.5	46.4	
Chemotherapy (%)			
Received	4.5	5.2	0.656
Chemotherapy regimen (%)			0.841
No chemotherapy	95.5	94.8	
Chemotherapy only	2.3	2.9	
Chemotherapy and surgery	2.3	2.3	
Radiation (%)	48.5	46.1	0.516
Radiation regimen (%)			0.809
No radiation	51.5	53.9	
Radiation only	14.0	13.4	
Radiation and surgery	34.4	32.7	
Radiation sequence (%)			0.011 *
Before	13.2	7.9	
After	82.0	77.2	
Other sequence	4.8	14.9	

* statistically significant.

**Table 3:** Vertebral column survival.

Variable	Male (*n* = 176)	Female (*n* = 106)	*p*-Value
5-Year Survival (%)	33 (25, 40)	41 (31, 51)	0.220
Median Survival (Months)	106 [87, 139]	83 [58, 119]	0.230

**Table 4:** Hazard ratio—vertebral column.

Variable	Hazard Ratio	95%CI		*p*-Value
		2.5%	97.5%	
Age	1.00	0.99	1.01	0.783
Sex				
Male	Reference			
Female	1.20	0.89	1.63	0.239
Race				
Non-Hispanic White	Reference			
Non-Hispanic Black	1.54	0.71	3.30	0.272
Hispanic	1.24	0.78	1.95	0.362
Other	1.72	0.97	3.06	0.065
Tumor Size	1.00	0.99	1.00	0.440
Surgery	1.06	0.68	1.64	0.796
Chemotherapy	0.92	0.41	2.08	0.841
Radiation	1.14	0.83	1.55	0.414

**Table 5:** Pelvis survival.

Variable	Male (*n* = 176)	Female (*n* = 106)	*p*-Value
5-Year Survival (%)	33 (27, 40)	37 (28, 46)	0.820
Median Survival (months)	92 [78, 107]	85 [69, 116]	0.820

**Table 6:** Hazard ratio—pelvis.

Variable	Hazard Ratio	95%CI		*p*-Value
		2.5%	97.5%	
Age	1.00	0.99	1.01	0.652
Sex				
Male	Reference			
Female	0.97	0.74	1.26	0.816
Race				
Non-Hispanic White	Reference			
Non-Hispanic Black	1.30	0.75	2.24	0.356
Hispanic	2.06	1.42	2.99	<0.001 *
Other	1.61	1.09	2.38	0.016 *
Tumor Size	1.00	1.00	1.00	0.555
Surgery	0.73	0.54	0.97	0.031 *
Chemotherapy	0.92	0.47	1.79	0.803
Radiation	1.25	0.96	1.63	0.092

* statistically significant.

## Data Availability

The data were obtained from the National Cancer Institute and are available from the authors with the permission of the National Cancer Institute.

## Abbreviations

The following abbreviations are used in this manuscript:

SEER	Surveillance, Epidemiology, and End Results
ICD-O	International Classification of Diseases for Oncology
WHO	World Health Organization
GTR	gross total resection
SD	standard deviation
IQR	interquartile range
ANOVA	analysis of variance
KM	Kaplan–Meier
HR	hazard ratio
CI	confidence interval
IRR	incidence rate ratio
